# DAILY CONNECTIONS BETWEEN HAPPINESS, SUBJECTIVE AGE, AND SUBJECTIVE ECONOMIC STATUS IN TURKIYE

**DOI:** 10.1093/geroni/igae098.1735

**Published:** 2024-12-31

**Authors:** Reyyan Can, Shevaun Neupert

**Affiliations:** North Carolina State University, Raleigh, North Carolina, United States; North Carolina State University, Raleigh, North Carolina, United States

## Abstract

Subjective economic status, which includes feelings of financial strain, is linked with important health and well-being indicators such as happiness. In addition, threats to economic status may be especially harmful for middle-aged and older adults. We aimed to characterize and explore predictors of daily happiness among middle-aged and older adults in Turkiye. Conducted during the summer of 2022, a period marked by high inflation, the study involved 98 participants aged 50 to 90 (M = 59.57, SD = 7.8) who reported on their happiness, subjective economic status, and subjective age for 14 consecutive days. Results from Multilevel Modeling revealed significant variations in happiness, subjective economic status, and subjective age over the 14-day period at both within and between person levels. Increases in subjective economic status were associated with increased daily happiness, whereas feeling older corresponded to decreased happiness. Subjective economic status and subjective felt age together accounted for 44% of the variance in within-person happiness variability, underscoring the importance of these factors in shaping happiness. Moreover, our study highlights economic volatility experienced by individuals in Turkiye calling for attention from policymakers, practitioners, and stakeholders to address the vulnerabilities faced by this group.

